# Supplementary material for: A fault-intrusion-tolerant system and deadline-aware algorithm for scheduling scientific workflow in the cloud
Source: PeerJ Comput Sci. 2021 Nov 2;7:e747. doi: 10.7717/peerj-cs.747 (PMC8576554; doi:10.7717/peerj-cs.747)
Supplement: Supplemental Information 6 [file peerj-cs-07-747-s006.zip › Myproject1.1/docs/org/cloudbus/cloudsim/class-use/File.html]

Uses of Class org.cloudbus.cloudsim.File


JavaScript is disabled on your browser.


- Overview
- Package
- Class
- Use
- Tree
- Deprecated
- Index
- Help

- Prev
- Next

- Frames
- No Frames

- All Classes

## Uses of Class org.cloudbus.cloudsim.File

- Packages that use File

  | Package | Description |
  | --- | --- |
  |  |  |
  | --- | --- |
  | org.cloudbus.cloudsim |  |
  | org.workflowsim |  |
  | org.workflowsim.clustering |  |
  | org.workflowsim.scheduler |  |
  | org.workflowsim.utils |  |
- - ### Uses of File in org.cloudbus.cloudsim

    Methods in org.cloudbus.cloudsim that return File

    | Modifier and Type | Method and Description |
    | --- | --- |
    |  |  |
    | --- | --- |
    | `File` | HarddriveStorage.`deleteFile(java.lang.String fileName)` Removes a file from the storage. |
    | `File` | Storage.`deleteFile(java.lang.String fileName)` Removes a file from the storage. |
    | `File` | HarddriveStorage.`getFile(java.lang.String fileName)` Gets the file with the specified name. |
    | `File` | Storage.`getFile(java.lang.String fileName)` Gets the file with the specified name. |
    | `File` | File.`makeMasterCopy()` Clone this file and make the new file as a **master** copy as well |
    | `File` | File.`makeReplica()` Clone this file but the clone file is set to a **replica** |

    Methods in org.cloudbus.cloudsim with parameters of type File

    | Modifier and Type | Method and Description |
    | --- | --- |
    |  |  |
    | --- | --- |
    | `int` | Datacenter.`addFile(File file)` Adds a file into the resource's storage before the experiment starts. |
    | `double` | HarddriveStorage.`addFile(File file)` Adds a file to the storage. |
    | `double` | SanStorage.`addFile(File file)` Adds a file to the storage. |
    | `double` | Storage.`addFile(File file)` Adds a file to the storage. |
    | `double` | HarddriveStorage.`addReservedFile(File file)` Adds a file for which the space has already been reserved. |
    | `double` | SanStorage.`addReservedFile(File file)` Adds a file for which the space has already been reserved. |
    | `double` | Storage.`addReservedFile(File file)` Adds a file for which the space has already been reserved. |
    | `protected boolean` | Datacenter.`contains(File file)` Checks whether the resource has the given file. |
    | `boolean` | HarddriveStorage.`contains(File file)` Checks whether a certain file is on the storage or not. |
    | `boolean` | Storage.`contains(File file)` Checks whether a file is stored in the storage or not. |
    | `double` | HarddriveStorage.`deleteFile(File file)` Removes a file from the storage. |
    | `double` | SanStorage.`deleteFile(File file)` Removes a file from the storage. |
    | `double` | Storage.`deleteFile(File file)` Removes a file from the storage. |
    | `double` | HarddriveStorage.`deleteFile(java.lang.String fileName, File file)` Removes a file from the storage. |
    | `double` | SanStorage.`deleteFile(java.lang.String fileName, File file)` Removes a file from the storage. |
    | `double` | Storage.`deleteFile(java.lang.String fileName, File file)` Removes a file from the storage. |
    | `boolean` | HarddriveStorage.`renameFile(File file, java.lang.String newName)` Renames a file on the storage. |
    | `boolean` | Storage.`renameFile(File file, java.lang.String newName)` Renames a file on the storage. |

    Method parameters in org.cloudbus.cloudsim with type arguments of type File

    | Modifier and Type | Method and Description |
    | --- | --- |
    |  |  |
    | --- | --- |
    | `double` | HarddriveStorage.`addFile(java.util.List<File> list)` Adds a set of files to the storage. |
    | `double` | SanStorage.`addFile(java.util.List<File> list)` Adds a set of files to the storage. |
    | `double` | Storage.`addFile(java.util.List<File> list)` Adds a set of files to the storage. |

    Constructors in org.cloudbus.cloudsim with parameters of type File

    Constructor and Description

    `File(File file)`

    Copy constructor, i.e.
  - ### Uses of File in org.workflowsim

    Methods in org.workflowsim with parameters of type File

    | Modifier and Type | Method and Description |
    | --- | --- |
    |  |  |
    | --- | --- |
    | `void` | Task.`addFile(File file)` Adds a file to existing file list |
    | `void` | CondorVM.`addLocalFile(File file)` Adds a file to the local file system |
    | `boolean` | CondorVM.`hasLocalFile(File file)` Tells whether a file is in the local file system |
    | `void` | CondorVM.`removeLocalFile(File file)` Removes a file from the local file system |

    Method parameters in org.workflowsim with type arguments of type File

    | Modifier and Type | Method and Description |
    | --- | --- |
    |  |  |
    | --- | --- |
    | `protected double` | DatacenterExtended.`processDataStageIn(java.util.List<File> requiredFiles, Cloudlet cl)` |
    | `void` | Task.`setFileList(java.util.List<File> list)` Sets a file list |
  - ### Uses of File in org.workflowsim.clustering

    Methods in org.workflowsim.clustering that return types with arguments of type File

    | Modifier and Type | Method and Description |
    | --- | --- |
    |  |  |
    | --- | --- |
    | `java.util.List<File>` | BasicClustering.`getTaskFiles()` Gets the files |
    | `java.util.List<File>` | ClusteringInterface.`getTaskFiles()` get all the task files. |
  - ### Uses of File in org.workflowsim.scheduler

    Method parameters in org.workflowsim.scheduler with type arguments of type File

    | Modifier and Type | Method and Description |
    | --- | --- |
    |  |  |
    | --- | --- |
    | `protected double` | DataAwareScheduler.`dataTransferTime(java.util.List<File> requiredFiles, Cloudlet cl, int vmId)` |
  - ### Uses of File in org.workflowsim.utils

    Methods in org.workflowsim.utils that return File

    | Modifier and Type | Method and Description |
    | --- | --- |
    |  |  |
    | --- | --- |
    | `static File` | ReplicaCatalog.`getFile(java.lang.String fileName)` Gets the file object based its file name |

    Methods in org.workflowsim.utils with parameters of type File

    | Modifier and Type | Method and Description |
    | --- | --- |
    |  |  |
    | --- | --- |
    | `static void` | ReplicaCatalog.`setFile(java.lang.String fileName, File file)` Adds a file name and the associated file object |

- Overview
- Package
- Class
- Use
- Tree
- Deprecated
- Index
- Help

- Prev
- Next

- Frames
- No Frames

- All Classes
